# Study for Laser Controlled Fabrication of Micro/Nano-Structures of Silicon Based on Multi-Physics Model

**DOI:** 10.3390/mi12050528

**Published:** 2021-05-07

**Authors:** Liqun Wu, Jianlong Chen, Linan Zhang, Hongcheng Wang, Chao Chen

**Affiliations:** 1 School of Mechanical Engineering, Hangzhou Dianzi University, Hangzhou 310018, China; wuliqun@hdu.edu.cn (L.W.); chenjianlong@hdu.edu.cn (J.C.); zln@hdu.edu.cn (L.Z.); wanghc@hdu.edu.cn (H.W.); 2 School of Information and Electronic Engineering, Zhejiang Gongshang University, Hangzhou 310018, China

**Keywords:** micro/nano-structures, micro/nano-cantilever sensor, temperature field, diffuse interface model

## Abstract

This work proposes a detailed process of micro/nano-structure surface modification in relation to temperature field. In this paper, a femtosecond laser is used to induce the surface morphology of a silicon substrate. We provide a new method for the fabrication of a micro/nano-cantilever probe by controlling the aspect ratio of the silicon surface morphology. A computational method is used to investigate the mechanical behaviors of early perturbation to late-stage structure. A diffuse interface model is employed to describe the evolution and provide a general framework. The theoretical model of femtosecond laser control surface morphology is verified by the experiments. For systematic study, the model involves the interface energy and kinetics of diffusion. This method provides an effective way to improve the sensitivity of micro/nano-cantilever sensors.

## 1. Introduction

Laser-induced surface evolution is an illuminating phenomenon that occurs on silicon by the radiation of a femtosecond laser [1]. The surface evolution of a silicon substrate plays a key factor in the field of micro-fabrication, photons, and magnetic field production [2,3,4]. The controlled laser pulses irradiate the surface of a silicon substrate; when the laser heat approaches the silicon ablation threshold, the periodic micro-structures appear [2,5]. Silicon-cantilever is widely used in micro/nano-mechanical sensors because of its good performance [6,7,8]; a high-resolution micro/nano-cantilever capacitive electric field sensor is produced by converting the electric field into the bending of the cantilever. The principle of a micro/nano-cantilever as the temperature sensor is that the resonant frequency is changing as the temperature changes, which could be used to detect the temperature in chemical and biological specimens [7]. Micro/nano-cantilever sensors could also be used for the mechanical detection of weak magnetic signals, such as the signals generated by nuclear or electron spins [8]. In the fabrication of the cantilever sensor, femtosecond laser processing is a common method employed to fabricate the surface of the cantilever. Femtosecond lasers can be used to induce multi-patterns on a variety of substrates, which may produce gradient surfaces with high stability, durability, and arbitrary shapes [9]. Currently, femtosecond laser processing is also used to construct asymmetrical surface wettability on both sides of the membrane [10]. In recent years, there has not been a remarkable breakthrough in research on the surface evolution of silicon because it is primarily limited by the complex experimental work and lack of effective numerical models [11,12,13]. Thus, we focus our effort on the study of the fabrication of a silicon cantilever.

In the experiments of laser-controlled surface evolution, many researchers focus on observing the evolution of micro-structures by changing laser parameters, such as laser wavelength and laser polarization [14,15,16]. However, the cost of these experiments and time-consuming processes limit further study of the fabrication of micro/nano-structures. Considering the shortcomings of experiments, many researchers make more effort to study the changes of the morphology. Numerical models, as an ideal approach, could be used to test the evolution of micro/nano-structures more clearly [17,18]. The previous studies represented the underlying mechanism of laser-controlled surface evolution by using the one-dimensional or two-dimensional models [19,20,21]. The energy of the laser is transferred to thermal energy, which causes mass transport on the silicon surface. The change in local temperature field drives thermal diffusion and causes the morphological evolution changes on the surface. Nevertheless, the current research is limited, and it is difficult to correctly formulate and predict the shape of modified surfaces based on their database.

Here, we propose a three-dimensional dynamic model. The model is developed based on a phase field model, which incorporates multi-energies and dynamics, and presents the morphological evolution process of micro/nanoscale structures. We employ this computational model to design the desired structures, and we systematically study the effect of the laser power and the thermal conductivity of the materials on the preparation process. Finally, a high aspect ratio probe for the micro/nano-cantilever sensor is prepared.

## 2. Materials and Methods

In order to develop a three-dimensional dynamic model, a phase field model was employed. Phase field models may provide reliable efficiency on modeling the evolution of micro/nano-structures [22,23]. Many morphological evolutions of the microstructure system have been represented based on the diffuse interface model. The significance of the mathematical modeling in understanding the relationship between the initial shape and the fabricated structure on the surface of silicon has been generally recognized.

**Figure 1:**
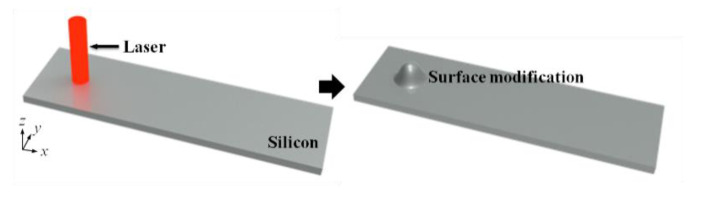
Schematic drawing represents laser irradiating the top surface of the silicon substrate.

A conserved variable c (x,y,z,t) was used to describe the morphological evolution of silicon. A schematic drawing in Figure 1 represents laser irradiating the top surface of silicon. After laser irradiation, the absorbed heat developed a temperature gradient throughout the substrate. The temperature gradient induced the thermophoresis force, which could affect the mass transport of atoms on the silicon surface. We define c as the mass fraction of silicon; C = 1 for a silicon phase and C = 0 for an air phase. We regard c (x,y,z,t) as a time-dependent and spatially continuous function. The thermal conductivity of silicon is 150 W/m-K. The surface diffusion coefficient of silicon increases as the temperature increases [24]. Correspondingly, the minimum value of the surface diffusion coefficient for the evolution was $0.5\times 10^{-5}\;\mu m^{2}/s$ at 450 °C, and the diffusion coefficient was approximately $1.0\;\mu m^{2}/s$ at 1200 °C.

A cantilever was used in the experiment to modulate the surface with the laser. Laser power was approximately 75 mW and wavelength (λ) was 532 nm, which was exposed to the cantilever for 10 s. When the laser irradiates the top surface of silicon, this causes mass transport on the surface, and the growth mechanism was first noted by Ivan Stranski and Lyubomir Krastanov [25,26,27,28]. The total free energy of the whole system is defined by following the Cahn–Hilliard model [29]. The morphological shape on the surface of silicon in the experiment was observed under an atomic force microscope (AFM). AFM images are used to give detailed pictures of bumps at different positions.

(1) $$G=\int_{V}\left[f(c)+\;\frac{1}{2}h{\left(\nabla c\right)}^{2}\right]dV$$

The first term represents the chemical energy that drives phase separation. We use a double-well function, $f(c)=f_{0}c^{2}{\left(1-c\right)}^{2}$, where $f_{0}$ is a positive constant. The remaining term illustrates the surface energy of the material, and h is a gradient energy coefficient. A driving force for mass transport is attributed to the chemical potential, $F_{d}=\;-\nabla \mu$. The chemical potential is defined by μ= δG/δc, where δG/δc is related to the total free energy of the system.

During laser irradiation, the substrate is placed in a temperature field gradient. The mass flux can be rewritten as $J_{c}=-\varepsilon Mc\nabla T$. Here, the constant ε is equal to 2/a and $M=Da/2k_{B}T$ [30]. D is the thermal diffusion coefficient, $k_{B}$ is the Boltzmann constant, and a is interface area. The total net flux could be expressed as J= −M∇μ−εMc∇ T. We note M(c) and the mobility will vanish outside the interfacial region of C [23,31,32].

(2) $$M(c)=M_{0}[(\int c^{2}{\left(1-c\right)}^{2}dc/\int_{0}^{1}c^{2}{\left(1-c\right)}^{2}dc)(1-c)]$$

The evolution of the variable C is governed by the Cahn–Hilliard nonlinear diffusion equation and combined with the mass conservation relation, $\frac{\partial c}{\partial t}=\;-\nabla \cdot J$. The expression is normalized with a characteristic length $L_{c}$, time $t_{c}\;=\;L_{c}^{2}\;/M_{0}f_{0}$, and the constant $\alpha =\varepsilon /f_{0}$ as follows:

(3) $$\frac{\partial c}{\partial t}=-\nabla \cdot (-M\nabla \mu_{1})$$

where $\mu_{1}=[4c^{3}-6c^{2}+2c-ch^{2}\nabla^{2}c+\alpha cT]$. The significance of the surface energy of material is described by the Cahn number, $ch^{2}\;=\;h\;/L_{c}^{2}f_{0}$. The temperature field generated by the laser is calculated by solving the heat diffusion equations [23,25].

(4) $$\rho \lambda \frac{\partial T}{\partial t}=k(\frac{\partial^{2}T}{\partial x^{2}}+\frac{\partial^{2}T}{\partial y^{2}}+\frac{\partial^{2}T}{\partial z^{2}})+Q$$

The term of Gaussian heat source could be solved by $Q=(1-R)I_{0}\beta \exp(-\beta {\left(x^{2}+y^{2}+z^{2}\right)}^{\frac{1}{2}})$ [25]. Here, the laser intensity is defined as $I_{0}(x,y,z)=\frac{2*P}{\pi \sigma^{2}}\exp(-\frac{2(x^{2}+y^{2}+z^{2})}{\sigma^{2}})$ [25,26]. P is the laser power and σ is the radius of the laser beam. A semi-implicit Fourier spectral method and semi-implicit backwards differentiation formula scheme are employed for the computational efficiency and stability [33,34,35]. To consider the variable mobility, we rewrite the right-hand side of Equation (2) as

(5) $$\nabla \cdot (M\nabla \mu_{1})\;=\;A\nabla^{2}{\mu_{1}}^{\prime }\;+s_{\mu_{1}}$$

where ${\mu_{1}}^{\prime }$ is the linear component of $\mu_{1}$. $s_{\mu_{1}}\;=\;\nabla \cdot (M\nabla \mu_{1})\;-\;A\nabla^{2}{\mu_{1}}^{\prime }$. Linear term $A\nabla^{2}{\mu_{1}}^{\prime }$ is implicitly treated and nonlinear term $s_{\mu_{1}}$ is explicitly treated with the semi-implicit Fourier spectral method. The linear component is considered as ${\mu_{1}}^{\prime }\;=\;2c\;-\;ch^{2}\nabla^{2}c$ for numerical stability. Combined with the semi-implicit Fourier spectral method and the SBDF time integration scheme, we obtain the following discretized form:

(6) $$\frac{3}{2}c^{n+1}-2c^{n}+\frac{1}{2}c^{n-1}=A\Delta t(2\tau \nabla^{2}c^{n+1}-ch^{2}\nabla^{4}c^{n+1})+2P^{n}-P^{n-1}$$

In the above function, the number of A=1 and $P^{n}\;=A\Delta t[\nabla \cdot (M\nabla {\mu_{1}}^{n})-2\tau \nabla^{2}c^{n}+\tau ch^{2}\nabla^{4}c^{n}]$. The temperature field represented by Equation (4) is calculated by a finite difference method. We apply the Fourier transform to Equation (6), and then the transformed equation is as follows:

(7) $${\hat{c}}^{n+1}=\frac{1}{Dev}(4{\hat{c}}^{n}-{\hat{c}}^{n-1}+4{\hat{P}}^{n}-2{\hat{P}}^{n-1})\;$$

here, $Dev=3+4\Delta t\tau k^{2}+2\Delta t\tau ch^{2}k^{4}$. The caret ‘∧’ and the subscript k stand for the Fourier transform.

## 3. Results and Discussion

**Figure 2:**
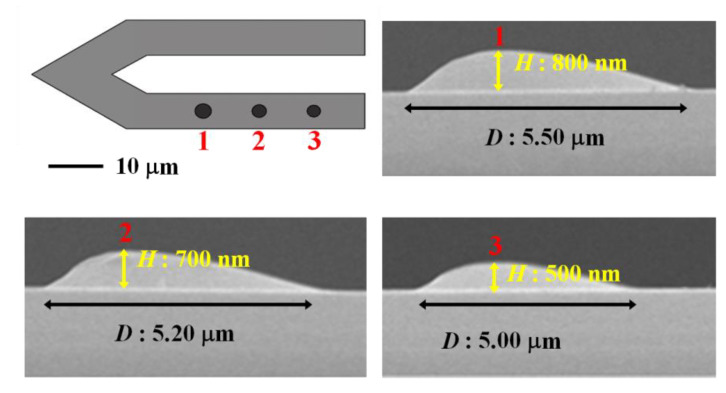
Atomic force microscope (AFM) images of surface modification at different positions: 1–3.

**Figure 3:**
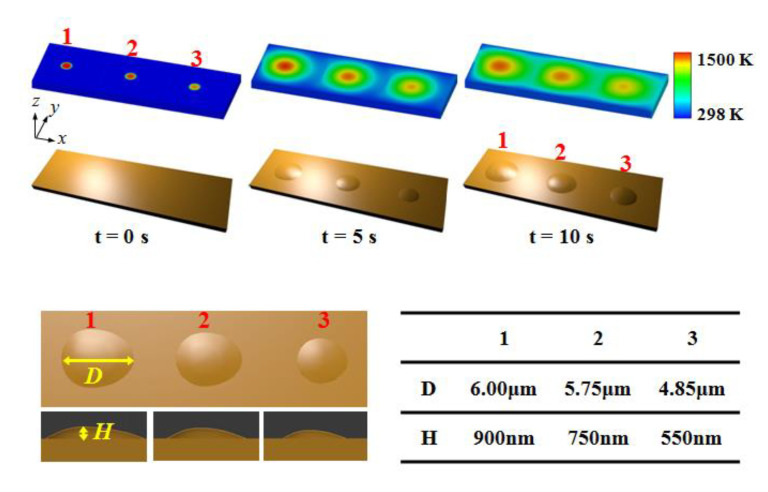
Simulated results of calculated temperature field and surface modification at different positions: 1–3.

In the experiment, the femtosecond laser (power approximately 75 mW, wavelength 532 nm, the repetition frequency 1 kHz, and the pulse width 120 fs) irradiated directly on the surface of cantilever; the spot diameter of the collimated laser was 8 μm, which was exposed to the cantilever for 10 s. In Figure 2, a detailed graph of the bumps is presented at different positions, 1–3, by using AFM images. The surface modification was more visible (the modified height increases) when the laser irradiated on the free end of the cantilever. The free end of the cantilever was in contact with air. The other end of the cantilever is fastened to the fixture with the same material as the cantilever. The observations of the simulated works are represented from t = 0 s to t = 10 s in Figure 3. To simulate multi-bumps growing on the surface, heat sources were given at position 1, 2, and 3. The domain size of the model was selected to be $10.0\times 30.0\times 1.5\;\mu m^{3}$ and the characteristic length was taken to be 50 nm. These quantitative observations indicated a good agreement between the experimental and simulated results.

In the model, the distance between the neighboring heat sources (laser spots) was approximately 10 μm in the cantilever. The thermal conductivity of silicon, 150 W/m-K, was applied at the end of the right domain to represent that the cantilever was in contact with the same material on the right side. At the left side of the domain, there were two different boundary conditions in the model. The air convection boundary condition was employed to indicate that the cantilever was in contact with the air; the thermal conductivity of air is approximately 0 W/m-K. Another boundary condition is the constant surface heat flux boundary condition, which was used to simulate the same material silicon between the neighboring laser spots in the cantilever.

### 3.1. Effect of Laser Power on Surface Modification

**Figure 4:**
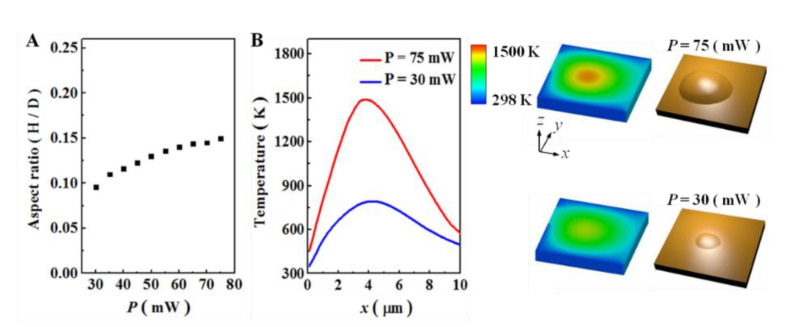
(**A**) Aspect ratio of bump with various laser powers. (**B**) The computational temperature gradients and morphological shape of bumps at laser powers of 75 mW and 30 mW. Higher laser power induces a distinct temperature gradient and morphological shape of the bump on the surface of the substrate.

In order to design the different morphological shapes of the bump with the laser, the effect of various laser powers was introduced by using a computational model. Enhancing the laser power from 30 mW to 75 mW, we observed that the highest point of each bump increased from 250 nm to 900 nm. The relationship between the aspect ratio of the bump and the variation of the laser powers, P, is plotted in Figure 4A. The aspect ratio of the bump increased as laser power increased. Since the laser power was enhanced from 30 mW to 75 mW, the aspect ratio of the bump increased by 56% (from 0.096 to 0.150). To clearly examine the influence of different laser powers on the temperature field in affecting surface modification, two surface modifications with different laser powers of 75 mW and 30 mW were represented. As shown in Figure 4B, a larger laser power created a rapid temperature jump, which means a large temperature gradient in the same localized surface region was generated.

In both cases, the highest temperature was 1500 K or 795 K when the laser power was 75 mW or 30 mW, respectively. The temperature gradient is considered as the only major external force to induce atom migration. The computational study of the effect of the laser power on surface modification suggests that the aspect ratio of the bump could be controlled by proper laser parameters. The investigation gives a unique relation between the temperature gradient and the variational laser power to estimate surface modification based on our computational method. Computational analysis is important to estimate the effect of thermal properties on surface modification

### 3.2. Effect of Thermal Conductivity of Surrounding Material on Surface Modification

**Figure 5:**
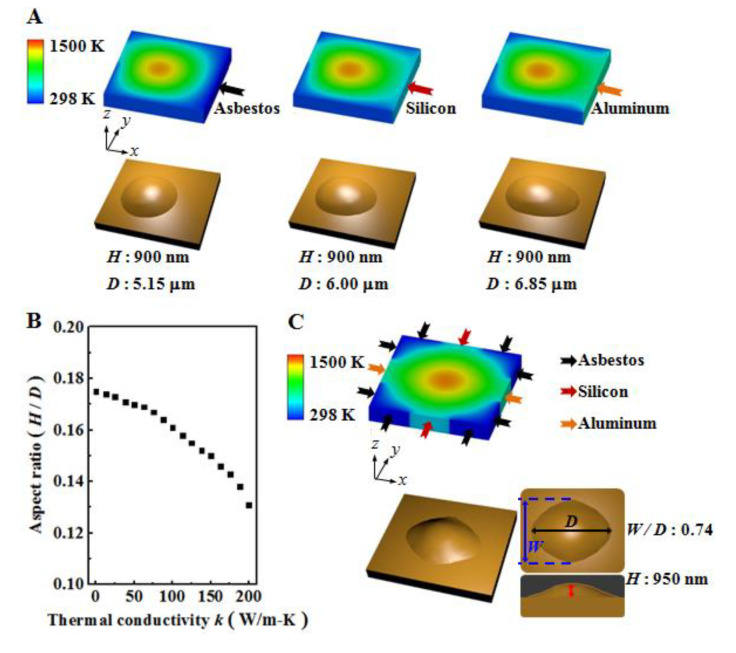
(**A**) Three different surrounding materials have been assigned at the right end of the domain. The different surrounding materials: non-conducting material (asbestos), same material (silicon), high-conductivity material (aluminum). (**B**) Aspect ratio of the bump with various thermal conductivities of surrounding materials (asbestos: 0 W/m-K; silicon: 150 W/m-K; aluminum: 200 W/m-K). (**C**) A designed structure is performed by connecting different materials (with different thermal conductivities) at the surrounding area of the substrate.

In Figure 5A, three models with the different boundary conditions at the end of the right side have been performed to indicate the cantilever connecting with the different materials: non-conducting material (thermal conductivity 0 W/m-K, asbestos), same material (thermal conductivity 150 W/m-K, silicon), and high-conductivity material (thermal conductivity 200 W/m-K, aluminum). In each case the highest point of the bump was 900 nm. However, thermal conductivity equal to 200 W/m-K on the right end promoted the temperature transferring more easily and quickly than other cases, which induced a wider distribution of the bump, 6.85 μm along the temperature transferring direction. Meanwhile, the width of the bump was 6.00 μm when the thermal conductivity was 150 W/m-K. The thermal conductivity of non-conducting material, 0 W/m-K, at the right end indicated that the width of the bump was 5.15 μm. The aspect ratio of the bump in the case of surrounding materials with lower thermal conductivity, 0 W/m-K, was 0.175. In the other two cases, the aspect ratio of the bump was 0.150 and 0.131, respectively. The details are shown in Figure 5B. The aspect ratio of the bump decreased as the thermal conductivity of the surrounding material increased. The qualitative observation of the morphological changes on the surface demonstrates that a higher aspect ratio of the bump could be obtained when the surrounding material has low thermal conductivity.

After considering the effect of the surrounding material on the aspect ratio of the bump, the next step was to design the resultant morphology by using different surrounding materials. As shown in Figure 5C, the three-dimensional structure with protrusions in different directions was developed based on the designed surrounding materials. The different thermal conductivity of the surrounding material determines the different gradient of temperature needed to influence the shape of the bump. We measured the ratio between the width of bump *W* and *D* as 0.74. The quantitative analysis of the surface modification due to the designed conditions provides a significant contribution to driving the fabrication process. This effort focuses on a fabrication technique for micro/nanoscale various surface features on the materials.

## 4. Conclusions

Our work presents a simple and efficient technique to fabricate micro/nanoscale bumps on a silicon surface. The quantitative observations provide guidance on predicting the modifications by some design works. The phase field model is introduced to study the fabrication of the surface of silicon controlled by a femtosecond laser; the fabricated surface is employed to be the probe of a micro/nano-cantilever. The theoretical analysis looks at various challenges such as analyzing the morphology, and measuring the size of the bump with respect to laser power and thermal conductivity. Femtosecond laser induced silicon surface morphology is an effective way to fabricate a micro/nano-cantilever. The innovation of our work indicates a new method for predicting surface modification and controlling the surface morphology by changing the aspect ratio of the silicon surface, and our work provides a reliable method for the preparation of high-quality micro/nano-cantilever sensors.
